# Nix Protein Positively Regulates NF-κB Activation in Gliomas

**DOI:** 10.1371/journal.pone.0044559

**Published:** 2012-09-12

**Authors:** Yuntao Lu, Leyu Wang, Minyi He, Wenhua Huang, Hong Li, Yongkui Wang, Jiming Kong, Songtao Qi, Jun Ouyang, Xiaozhong Qiu

**Affiliations:** 1 Department of Anatomy, Key Laboratory of Construction and Detection of Guangdong Province, Southern Medical University, Guangzhou, Guangdong, China; 2 Department of Neurosurgery, Affiliated Nangfang Hospital, Southern Medical University, Guangzhou, Guangdong, China; 3 Department of Organ Transplantation, Zhujiang Hospital, Southern Medical University, Guangzhou, Guangdong, China; 4 Department of Human Anatomy and Cell Science, University of Manitoba, Winnipeg, Manitoba, Canada; University of Chicago, United States of America

## Abstract

Previous reports indicate that the *NIX/BNIP3L* gene acts as a pro-apoptotic factor by interacting with *BCL2* and *BCL-XL*, playing an important role in hypoxia-dependent cell death and acting as a tumor suppressor. However, many studies also showed that *NIX* is linked to a protective role and cell survival in cancer cells. Nuclear factor-κB (NF-κB) can attenuate apoptosis in human cancers in response to chemotherapeutic agents and ionizing radiation. We observed an absence of i-κBα (NF-κB activation inhibitor) expression, but a greater expression of Nix and p-NF-κB proteins in the Nix-wt U251 cells, which was not observed in the Nix-kn cells under hypoxic conditions. Using electrophoretic mobility shift assay (EMSA) and luciferase detection, the activation of NF-κB was detected only in the Nix-wt U251 cells with hypoxia. These data imply that Nix protein might play a role in the positive regulation of the NF-κB pathway. Moreover, 46 cases of glioma also showed high levels of Nix protein expression, which was always accompanied by high p-NF-κB expression. Patients with Nix (+) showed less tissue apoptosis behavior in glioblastoma (GBM), unlike that observed in the Nix-negative patients (−). The same apoptotic tendency was also identified in anaplastic astrocytoma (AA) groups, but not in astrocytoma (AS). On analyzing the Kaplan-Meier curve, better tumor-free survival was observed only in cases of astrocytoma, and not in AA and GBM. Thus, our study indicates that Nix protein might have multiple functions in regulating glioma behaviors. In the low-grade gliomas (astrocytoma) with low expression of NF-κB, the cell death-inducing function that occurs through a Bax mechanism might predominate and act as a tumor suppressor. While in the malignant gliomas (AA and GBM), with higher expression of the *NIX* gene and with activity of the NF-κB pathway, the oncogene function of Nix was predominant.

## Introduction

As the most frequent type of malignant intracranial tumor, accounting for around 70% of primary central nervous system tumors, glioma is categorized into Grades II, III, and IV (WHO, 2007) according to the pathological malignancy [1]. Despite the wide usage of multi-therapeutic strategies, such as radiology plus concomitant and adjuvant chemotherarpy after surgery, the clinical outcome and prognosis of glioma patients remain extremely poor. The radio-chemotherapy program only improves the limited median survival period of Glioblastoma multiform (GBM) patients.

Among the varying elements, accumulating evidence indicates that dysfunctional apoptosis and consequent resistance to chemotherapy and radiotherapy closely correlates with tumorigenesis and poor prognosis [2]–[4]. So far, several cellular signaling pathways have been known to play roles in the anti-apoptosis of glioma cells, such as PTEN/PI3K/Akt, mTOR, and nuclear factor-κB (NF-κB) [5], [6]. NF-κB, a member of the Rel family of transcription factors, mediates apoptotic signaling pathways in various types of human cancers [7]–[9]. NF-κB can attenuate apoptosis in response to chemotherapeutic agents and ionizing radiation [10]. Therefore, the relationship between the NF-κB signaling pathway and gliomagenesis was studied. The data indicate that NF-κB can be upregulated and activated in gliomas [11], and its expression correlates with tumor grade and prognosis [12], [13].

Bnip3L, a BH3-only member of the Bcl-2 family also known as Nix, is induced by hypoxia [14], interacts with Bcl-2 and Bcl-xl, and acts as a pro-apoptotic factor through its C-terminal transmembrane domain [15], [16]. Nix expression induced by hypoxia retards cancer cell growth [17], [18]. The severity of tumor hypoxia strongly correlates with malignancy and WHO grade. Previous reports implied that Bnip3L/Nix play important roles in hypoxia-dependent cell death and act as tumor suppressors [15], [18], [19]. Striking upregulation of the *BNIP3L/NIX* gene products by hypoxia is observed in clinical tumors. Levels of NIX and BNIP3 mRNA were higher in the tumor samples compared to the normal samples in 5/5 and 3/5 cases, respectively [18]. Nix also functions as an effector of Gq-dependent cardiomyopathy, and negatively regulates tumor growth in nude mice injected with U2OS osteosarcoma cells [20]–[22]. Since both high expression of NF-κB and Nix occurred in gliomas, we tried to find the correlation between them in glioma specimens. In this study, the Nix knockdown U251 stable cell line was established and used to detect the expression of NF-κB pathway-related proteins. Several experiments in vitro were conducted to observe the cellular behavior under conditions of hypoxia and normoxia. In addition, 18 cases of astrocytoma (AS), 12 cases of anaplastic astrocytoma (AA), and 16 cases of glioblastoma (GBM) were taken to do related detections. To validate data from cells, the clinical outcomes of glioma patients were also reviewed and analyzed.

## Results

### Nix Protein Positively Regulates NF-κB Pathway Activation in vitro

**Figure 1:**
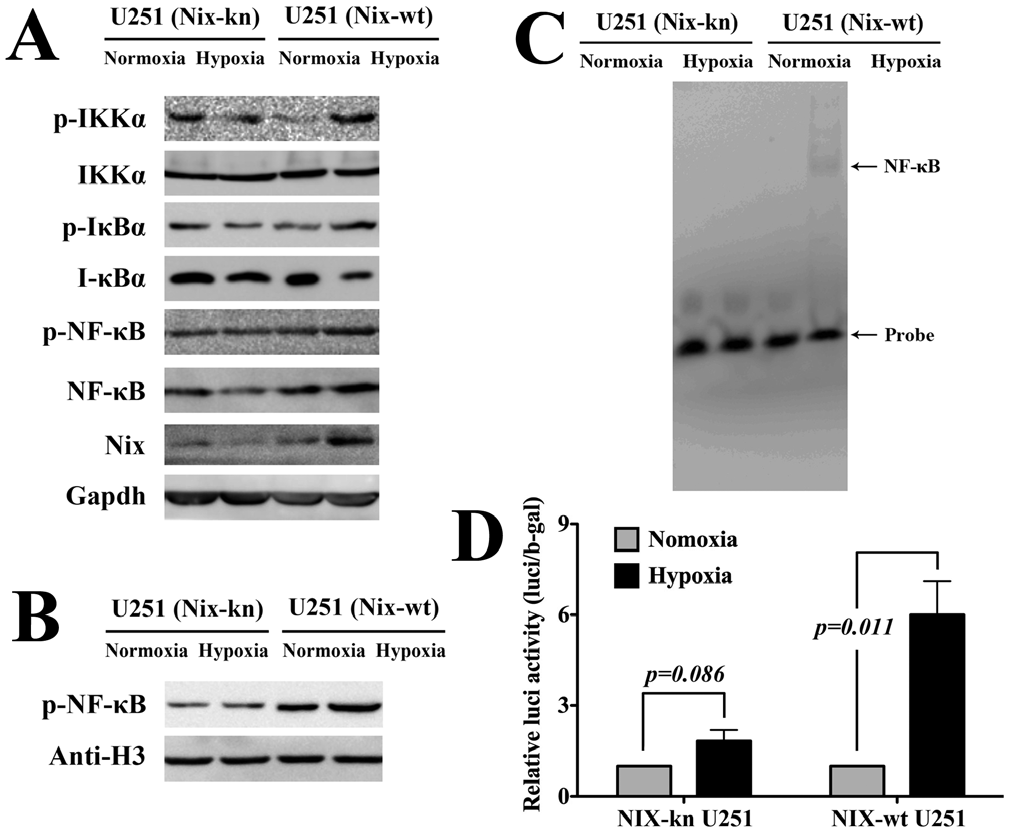
Nix protein positively regulates NF-κB activation in vitro. (**A**) When Nix-wt and Nix-kn U251 glioblastoma cell lines were incubated in DMEM medium under hypoxic conditions (5% CO_2_+95% N_2_), western blots revealed that the levels of Nix, p-NF-κB, p-IKK, and p-I-κBα were induced while I-κBα was degraded in Nix-wt U251 glioblastoma cells, but not in Nix-kn U251 glioblastoma cells. (**B**) Nuclear p-NF-κB was detected by western blot. The level of p-NF-κB in the nuclei of Nix-wt U251 cells was significantly higher than that of Nix-kn U251 cells. (**C**) The activation of NF-κB detected by EMSA. The activation of NF-κB was only detected in the Nix-wt U251 cells subjected to hypoxia. (**D**) The luciferase detection of NF-κB activation (repeated in triplicate). The activation of NF-κB was only detected in the Nix-wt U251 cells subjected to hypoxia (p<0.01).

The protein expression levels of NF-κB, p-NF-κB, IκBα, p-IκBα, IKKα, and p-IKKα were tested in Nix knockdown (Nix-kn) and control (Nix-wt) U251 glioblastoma cell lines under conditions of normoxia and hypoxia. When the cells were incubated in DMEM medium under hypoxic conditions (5% CO_2_+95% N_2_), higher levels of Nix p-NF-κB/p65, p-IκBα, p-IKKα, and lower levels of IκBα were detected by western blot in Nix-wt U251 cells, but not in Nix-kn U251 cells (Figure 1A). The protein level of p-NF-κB in nuclei was also determined. The results showed a higher level of p-NF-κB in the Nix-wt U251 cells and nuclei subjected to hypoxia but not in the Nix-kn U251 cells (Figure 1B).

The activation of NF-κB was detected using an EMSA and luciferase reporter gene assay. The results showed that hypoxia activated NF-κB in the Nix-wt U251 cells but not in the Nix-kn U251 cells (Figure 1C, 1D). These data revealed that Nix protein acts as an important activator of the NF-κB pathway and contributes to cell recovery under oxygen-poor conditions.

**Figure 2:**
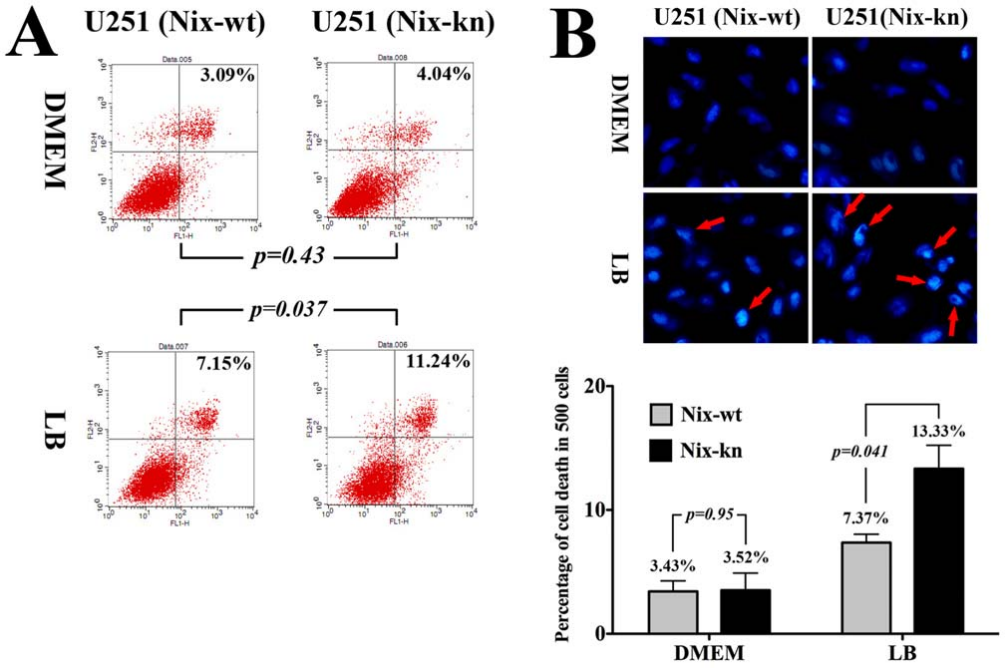
Nix protein improves cell survival in U251 cells cultured in nutritionally deficient conditions. (A) The apoptosis of Nix-wt and Nix-kn U251 cells with LB treatment and flow cytometry detection (Three repeated experiments). After LB medium was replaced with fresh DMEM +10% FBS medium, the apoptosis ratios for Nix-kn and Nix-wt U251 cells were 11.24±2.15% and 7.15±0.87% (*p* = 0.037), with corresponding controls of 4.04±0.89% and 3.09±1.65% (*p* = 0.43), respectively. (B) The viability of Nix-wt and Nix-kn U251 cells with LB treatment by DAPI. The dead cells are indicated by red arrows. Performed in triplicate, 500 cells were randomly selected for analysis, and the rate of dead cells for LB Nix-kn and LB Nix-wt U251 cells were 13.33±3.28% and 7.37±1.17%, with corresponding controls of 3.52±2.4% and 3.43±1.47%, respectively. The statistic data indicated significantly more death cells in the LB Nix-kn than Nix-wt cells (*p* = 0.041); while no difference was found between DMEM groups.

After Nix-wt and Nix-kn U251 cells were incubated in LB medium (10 g/L tryptone, 5 g/L yeast extract, and 10 g/L NaCl, pH 7.5) instead of the DMEM medium for 12 h, apoptosis was tested using flow cytometry (Annexin V-PI) and DAPI dye. After LB medium was replaced with fresh DMEM +10% FBS medium, more apoptotic cells were detected in LB Nix-kn cells than in LB Nix-wt cells, with apoptosis percentages of LB Nix-kn and LB Nix-wt U251 cells of 11.24±2.15% and 7.15±0.87%, respectively, compared with apoptosis in corresponding controls of 4.04±0.89% and 3.09±1.65%, respectively (Figure 2A). DAPI dye also showed more dead cells in Nix-kn U251 cells incubated in LB. Five hundred cells were randomly selected for analysis, and the rate of cell death for LB Nix-kn and LB Nix-wt U251 cells were 13.33±3.28% and 7.37±1.17%, and for the corresponding controls (DMEM cultured) were 3.52±2.4% and 3.43±1.47%, respectively (Figure 2B). Significant statistic difference was identified in the LB Nix-kn cells comparing to other groups by apoptosis (*p* = 0.037) and death cells (*p* = 0.041) detection, which also indicated that Nix-kn U251 cells have a more difficult time recovering than Nix-wt U251 cells do.

### The Role of Nix and p-NF-κB Expression in Clinical Glioma Samples

**Figure 3:**
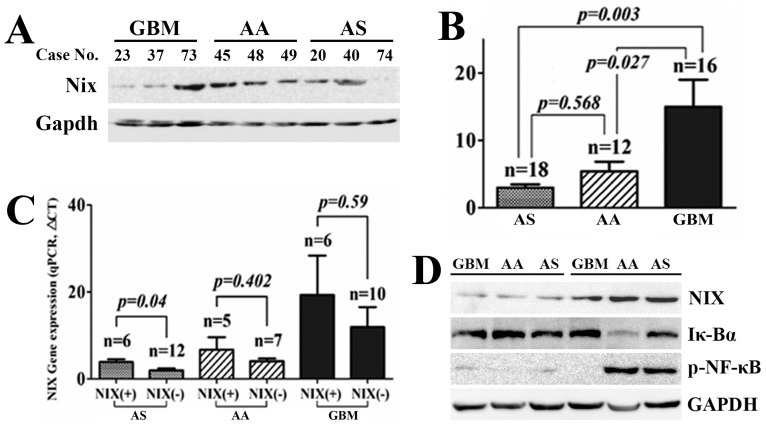
Nix protein and mRNA levels in 46 clinical glioma samples. (**A**) There was high expression of Nix protein (ratio of Nix/Gapdh >1) in 17 cases, referred to as Nix (+). In this figure, 4 cases of Nix (+) (cases No. 73, 45, 48, and 40) and 5 cases of Nix (−) (cases No. 23, 37, 49, 20, and 74) were observed according to the ratio of Nix/Gapdh. (**B**) The highest NIX mRNA levels were observed in GBM patients, compared to AS and AA patients. (**C**) Asynchronous expression of NIX mRNA and protein. A significant difference in Nix mRNA levels between Nix (+) and Nix (−) cases was seen in AS samples (*p = 0.04*), but not in grade III and grade IV patients. The results suggested that posttrancriptional regulation of the *NIX* gene predominantly existed in malignant gliomas. (**D**) High expression of Nix protein consistently accompanied high p-NF-κB and low I-κBα expression in glioma samples.

Nix protein level was evaluated by western blot in 46 glioma samples. Nix protein was more highly expressed (the ratio of Nix/Gapdh >1) in 17 cases, referred to as Nix (+), including 6 cases of AS (6/18, 33.3%), 5 of AA (5/12, 41.7%), and 6 of GBM (6/16, 37.5%) (Figure 3A). By quantitative Realtime PCR (qRT-PCR) detection, Nix mRNA was expressed at significantly higher levels in the GBM samples than in the AS and AA samples (*p* = 0.003 and 0.027, respectively; Figure 3B), but no difference was identified between AS and AA samples. When the samples were split into Nix (+) and Nix (−) subgroups, the significant difference in NIX mRNA levels between Nix (+) and Nix (−) was only seen in AA (*p = 0.04*), and not in AA and GBM samples (Figure 3C). The asynchronous expression of mRNA and protein suggested that posttranscriptional regulation most likely affected the Nix protein levels in malignant and high-grade gliomas (AA and GBM).

In addition, the high expression of Nix protein always accompanied high expression of p-NF-κB (NF-κB activation) in glioma samples of various pathologies (Figure 3D). According to the above data for glioblastoma cell lines and clinical samples, the expression of Nix protein probably functions by activating the NF-κB pathway in the tumorigenesis of gliomas.

### Correlation between Nix Protein Expression and Patient Prognosis

**Figure 4:**
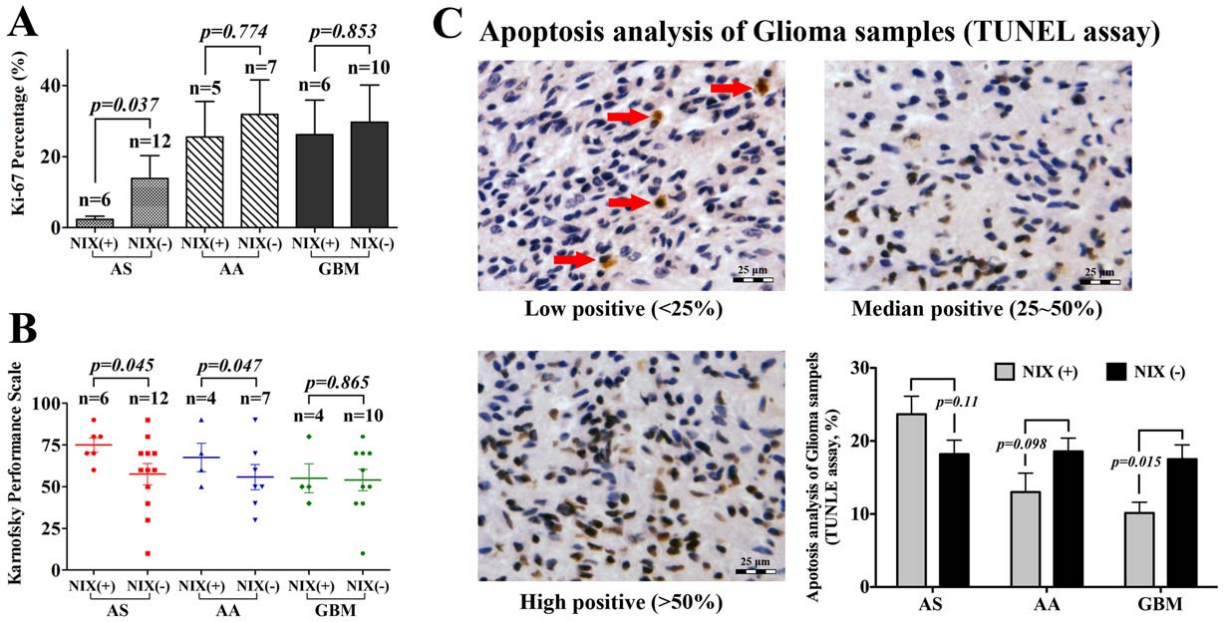
The influence of Nix protein on the behavior and prognosis of glioma patients. (**A**) In the 18 cases of astrocytoma, a high Ki-67 index indicated a more invasive ability in the Nix (−) cases (*p = 0.037*). (**B**) The KPS score showed that significant differences were found between Nix (+) and Nix (−) cases in astrocytoma (*p = 0.045*) and AA (*p = 0.047*). (**C**) Apoptosis analysis in 42 cases of clinical glioma samples by TUNEL assay, the result indicated that more severe tissue apoptosis happened in Nix (−) than Nix (+) GBM cases (*p* = 0.015). The same tendency was also displayed in AA samples, but not in AS groups, although the statistic significance was negative (*p* = 0.11 and 0.098).

In order to validate the role of Nix protein during tumorigenesis, the clinical data from glioma patients were reviewed and analyzed. The results of the Ki-67 index revealed that there was a significant difference between Nix (+) and Nix (−) in AS samples (*p = 0.037*), but not in AA and GBM samples, which indicated a different tumor invasive behavior in AS (Figure 4A). The clinical analysis also indicated better prognosis for Nix (+) AS cases, suggesting a tumor suppressor role of the *NIX* gene only in AS.

Moreover, the apoptosis analysis by TUNEL assay was used to further evaluate the biological function of Nix protein in 42 cases of clinical glioma samples. Between Nix (+) and (−) groups, significant different apoptosis percentage was only identified in GBM. Interestingly, more severe tissue apoptotic performance happened in Nix (−) samples rather than (+), which indicated contradict function to the pro-apoptotic factor of Nix protein (*p = 0.015*). However, no such difference was identified in AS and AA groups, although the same tendency could be found in AA.

**Figure 5:**
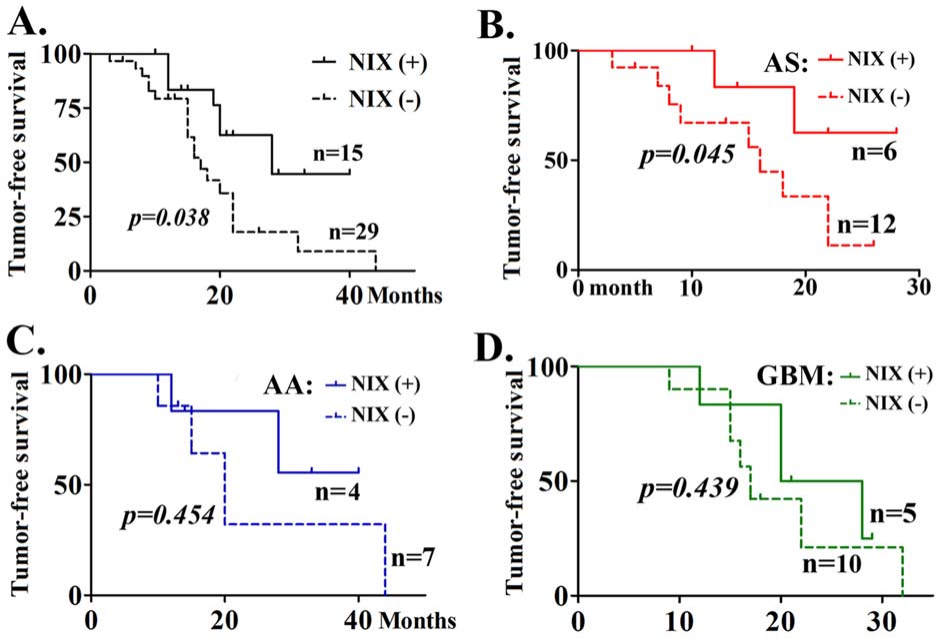
According to long-term follow-up of the Nix (+) and (−) groups of patients, tumor-free survival by Kaplan-Meier analysis criteria was significantly different in the total cases (*p = 0.038*) and in astrocytoma patients (Grade II) (*p = 0.045*), but not in AA (Grade III) and GBM (Grade IV) patients.

Tumor recurrence (Table 1) and the Karnofsky Performance scale (KPS) score were also determined (Figure 4B). After statistical evaluation of the KPS scores, a significant difference was found between Nix (+) and (−) groups for AS and AA (*p = 0.045* and *0.047*). Survival of the Nix (+) and Nix (−) groups of patients were determined by Kaplan-Meier analysis. The tumor-free survival curve consistently showed better prognosis for Nix (+) patients. However, if subdivided into tumors by grade, only in AS Nix (+) was the survival rate better than for Nix (−) patients; this was not true for AA or GBM (Figure 5).

**Table 1:** Tumor-related death and recurrence in 44 patients with glioma.

	AS (Grade II)		AA (Grade III)		GBM (Grade IV)	
	Nix (+)	Nix (−)	Nix (+)	Nix (−)	Nix (+)	Nix (−)
Death	1 (16.7%)	2 (16.7%)	1 (25%)	3 (42.9%)	1 (20%)	5 (50%)
Recurrence	1 (16.7%)	6 (50%)	1 (25%)	3 (42.9%)	3 (60%)	4 (40%)
Total (n)	6	12	4	7	5	10

## Discussion

Nix and its homologue Bnip3 (BCL2/adenovirus E1B 19 kD-interacting protein 3) are considered to be mitochondrial-localized members of the Bcl-2 family. Previous reports implied that Nix protein is a tumor suppressor in several kinds of cancers, and it acts by inducing cell death mainly through targeting mitochondria: directly through Bax- or Bak-dependent mechanisms, or indirectly through an effect on calcium stores in the endoplasmic reticulum [23]. The clonicity of cancer cells was significantly suppressed when cervical cancer cell lines were transfected with the *NIX* gene. There is a correlation between Nix and prostate cancer metastasis suppression [24]. The epigenetic silencing of Bnip3 in some colorectal cancers [25], [26] and in pancreatic cancers [27], suggests that Bnip3 is deleterious to tumor development/progression. However, there are also many studies that showed that Bnip3 and Nix had a protective role and promoted cell survival in cancer cells [14], [28], [29]. Both Bnip3 and Bnip3L/Nix mRNA levels are higher in breast cancer tissue than in normal tissue, and correlated with high-grade status and was associated with more invasive cases. All of the above research implies molecular regulation and a physiological function of Nix in tumor behavior is still far from clear.

In terms of glioma, although Nix can act as an effective tumor suppressor during early tumorigenesis of gliomas as previously reported [15], [18], [19], increased NIX mRNA levels may also be a molecular marker for the deterioration of glioma patients. The highest level of NIX mRNA appeared in Grade IV tumors among all the various types of glioma samples. Simultaneously, constitutive activation of NF-κB subunits is present in malignant astrocytoma, especially in GBM [30], and inhibition of NF-κB activation accelerates glioblastoma cell death [31]. The activation of NF-κB increases in gliomas as tumor grade increases. In this study, a correlation between Nix,NF-κB, and i-κBα (the inhibitor of NF-κB activation) was detected in the U251 cell line. By western blot, EMSA, and luciferase reporter gene assays, NF-κB was observed to be activated in Nix-wt U251 cells under hypoxic conditions, while the activation was weak in Nix-kn U251 cells under hypoxic conditions. The positive relationship between Nix and NF-κB activation and the inverse relationship between Nix and i-κBα protein levels indicated that Nix was an important activator of the NF-κB pathway. The LB incubation study also proved that Nix protein could act as an important activator of NF-κB activation, and consequently increase cell survival.

Our clinical study clearly showed that high expression of Nix proteins was always accompanied by high expression of p-NF-κB. A greater expression of Nix mRNA was observed in GBM (Grade IV) than in AA (Grade III) and AS (Grade II). This implied that Nix protein is not merely a tumor cell death-induced factor as proposed by previous studies [15], [18], [19], but that it can also positively regulate tumorigenesis through activating the NF-κB pathway. According to the differences in Nix expression by western blot analysis, the 46 glioma samples were divided into Nix (+) and Nix (−) groups. In AS, AA, and GBM patients, the prevalence of Nix (+) patients was 33.3%, 38.5%, and 40%, respectively, which indicated that differences in Nix expression happen frequently in glioma patients with various pathological grades.

In terms of clinical outcome, a significant difference in Ki-67 percentage between Nix (+) and Nix (−) patients was found only in Grade II astrocytoma (*p = 0.037*), which indicated a minor tumor invasion for NIX (+) patients and supported the tumor-suppressor role of the NIX gene. While such differences were not identified in AA (Grade III) and GBM (Grade IV) patients, this lead us to question if Nix might also have the inverse function of regulating cellular behaviors of malignant gliomas. Regarding the Kaplan-Meier analysis, only the AS Nix (+) survival rate was better than for Nix (−) patients, but not in AA or GBM, which also hinted at sophisticated functions of Nix protein as more than just a pro-apoptosis factor. More interestingly, by TUNEL assay, the tissue apoptotic percentage of clinical glioma samples was analysis and results showed that in GBM, less apoptosis tumor cells were identified in Nix (+) comparing to (−) groups. And in AA samples, the same tendency could also be identified, although the statistic significance was negative. Combined together with the apoptotic experiments in vitro, these results highly hinted that in malignant gliomas, Nix protein might play role to anti-apoptosis and act as oncogene function by activate NF-κB pathway.

Analyzing all of the above data, we proposed that it is most likely that Nix protein plays both positive and negative roles in the tumorigenesis of gliomas. In low-grade gliomas (Grade II) with relatively low expression of NF-κB, the cell death-inducing function through Bax mechanism might predominant, acting as a tumor suppressor. While in malignant gliomas (Grades III and IV), higher expression of the *NIX* gene and activity of the NF-κB pathway might promote the oncogene function. Furthermore, although different from the protein levels, there was no significant difference in NIX mRNA levels between the Nix (+) and Nix (−) samples in AA and GBM patients. The asynchronous levels of protein and mRNA expression suggested that posttranscriptional regulation of the *NIX* gene alters the Nix protein level, which should be further investigated in the future.

## Materials and Methods

### Cell Culture and Treatment

The U251 glioblastoma cell line (ATCC, USA) was cultured in DMEM supplemented with 10% heat-inactivated FBS, 100 U/mL penicillin and 100 µg/mL streptomycin under 5% CO_2_ conditions. Cell viability was determined by trypan blue exclusion. Both the shRNA (TCAACATCAAACATGATCTGC) used to knockdown Nix and the scramble shRNA (ACCTCATAACAAATTCTAGGC) were inserted into the “BLOCK-iT™ Lentiviral RNAi Expression System” (Invitrogen), according to the manufacturer’s instructions. The scramble shRNA was used for the negative control group. Briefly, the pLenti6/BLOCK-iT™ expression constructs were used to cotransfect the 293FT producer cell line and viral supernatant was harvested to transfect the U251 cells. Stable U251 cell sublines (Nix-kn and Nix-wt) were selected by Blasticidin. Following 12 h of hypoxic culture (5% CO_2_+95% N_2_), total proteins and nuclear proteins were extracted for western blot analysis, including nuclear proteins for Electrophoretic Mobility Shift Assay (EMSA). The cells were incubated in LB medium (10 g/L tryptone, 5 g/L yeast extract and 10 g/L NaCl, pH 7.5) followed by fresh DMEM +10% FBS medium, then detected by dapi dye and flow cytometry (PI/annexin V dyeing) to evaluate cell viability.

### Patients and Tissue Samples

Except for 26 cases excluded from a total 72 glioma patients because of death during the perioperative period or due to unavailability of tumor samples, 46 tumor specimens were obtained and approved pathologically between January 2007 and January 2009, including 18 cases of astroglioma (WHO Grade II), 12 cases of anaplastic glioma (AA, Grade III), and 16 cases of GBM (Grade IV). All the tissue samples were harvested by physicians from the tumor center. Patients with chemotherapy or radiotherapy before surgery were not recruited for this study. After surgical resection, the tumor tissues were immediately frozen and stored in liquid nitrogen until processing. Following the tumor resection, 28 patients with high-grade gliomas (Grades III and IV) received radiotherapy plus the concomitant and adjuvant temozolomide treatment [32].

This study was approved by the institutional review board of the Southern Medical University at Guangzhou, China. Related ethics approval had been obtained for this study from the ethics committee of the affiliated Nanfang Hospital, Southern Medical University. Every participant was notified and informed about the content of this study. Participants or their family members/informants signed written informed consent forms.

All patients underwent a long-term follow-up, except for 2 missed cases. The average follow-up period was 17.6±8.9 months (3–44 months). Tumor recurrence was determined by either magnetic resonance imaging (MRI) or the occurrence of new neurological symptoms. Clinical outcome information during the follow-up period was obtained by telephone or written correspondence and by reviewing the death certificate. The quality of patient life (QOL) was evaluated by the Karnofsky performance scale (KPS) score.

### Western Blot Analysis and Immunohistochemistry

The total proteins or nuclear proteins of cells or tissue were separated by performing SDS-PAGE after being boiled for 5 min, and then blotted onto a PVDF membrane. The primary antibodies were diluted in 5% (w/v) BSA in TBS and incubated overnight at 4°C. The membranes were incubated with horseradish peroxidase-conjugated anti-rabbit (1∶3,000) or anti-mouse (1∶3,000) antibodies for 1 hour at room temperature in 5% (w/v) milk powder in TBS-T buffer (10 mM Tris·HCl, pH 7.5, 500 mM NaCl, 0.05% Tween 20). Peroxidase activity was visualized with the Supersignal Chemiluminescent substrate (Santa Cruz), according to the manufacturer’s instructions. All results were evaluated compared to Gapdh. For the tissue samples, a Nix/Gapdh ratio >1 was defined as a Nix-positive sample (Nix (+)), while Nix/Gapdh <1 was defined as a Nix-negative sample (Nix (−)).

To evaluate the Ki-67 index, all clinical tumor samples were cut into 4 µm thick sections after fixation and embedded in paraffin. According to a routine procedure, the samples were incubated with a Ki-67 antibody (MIB-1, DakoCytomation, Glostrup, Denmark) at room temperature for 60 minutes. Following incubation with secondary antibody conjugated with streptavidin-biotin for 30 minutes, diaminobenzidine (Sigma, USA) was used as the chromogen. Results were evaluated by a neuropathologist using a DP-71 microscope (Olympus). The percentage of the Ki-67 positive region was determined by an average of 10 fields with high magnification (×400) with the software package Olysia Bioreport (Olympus Company, Japan).

In addition, exception of 4 cases because of sample volume and quantity, the other 42 cases of samples was used to do TUNEL assay (R&D, Minneapolis, USA). Staining was developed in freshly prepared diaminobenzidine solution (DAB, Sigma Co.) for 3–5 min, and then counterstained with hematoxylin, dehydrated, air dried, and mounted. The apoptotic percentage was determined by the same methods as Ki-67.

### Quantitative Real-time PCR Analysis

To validate the gene expression of NIX in all tumor samples, total RNA isolation and cDNA synthesis were done with TRIzol and Oligo dT (Invitrogen, USA). Quantitative real-time PCR (qPCR) was performed by SYBER Green assays (Applied Biosystems, USA). Amplification conditions were as follows: hold at 95°C for 10 minutes, followed by 40 cycles for 15 seconds at 95°C and 1 minute at 60°C. Thermal cycling and fluorescence detection were done using the StepOnePlus™ Real-Time PCR System (Applied Biosystems, USA). The NIX mRNA expression levels versus GAPDH were determined by the ΔCt method. The primers used for qPCR were as follows: Nix (±) gcagggaccatagctctcag/tgctcagtcgctttccaata and GAPDH (±) tgcaccaccaactgcttagc/ggcatggactgtggtcatgag.

### EMSA

The Biotin 3′ End DNA Labeling Kit and the LightShift Chemiluminescent EMSA Kit from Pierce Biotechnology (Rockford, IL) were used. The biotin-labeled NF-κB consensus oligonucleotide was as follows: 5′-agttgaggggactttcccaggc −3′ (Beyotime, China). This was performed according to the manufacturer’s instructions. The signals were documented with a CCD system.

### Luciferase Assay

The NF-κB-driven reporter plasmid was as described [33]. Transient transfection of the cells was performed using Lipofectamine 2000 from Invitrogen according to the manufacturer’s instructions. The relative luciferase activity was determined by measuring firefly luciferase activity and normalizing it to β-galactosidase activity.

### Statistical Analysis

All data are presented as means ± SD. Two independent samples t tests were used to compare the NIX mRNA expression and the Ki-67 index in tumor samples. The 2 independent samples nonparametric test was used to analyze the KPS scores. The Log-rank (Mantel-Cox) Test was used to evaluate statistical significance of tumor-free survival. All statistics were done with SPSS 15.0 software. The statistical significance was defined as a *p* value <0.05.
